# Awareness of nonalcoholic steatohepatitis and associated practice patterns of primary care physicians and specialists

**DOI:** 10.1186/s13104-016-1946-1

**Published:** 2016-03-11

**Authors:** Susan Polanco-Briceno, Daniel Glass, Mark Stuntz, Alexis Caze

**Affiliations:** Deerfield Institute, 780 3rd Ave., 36th Floor, New York, NY 10017 USA

**Keywords:** Nonalcoholic fatty liver disease, Nonalcoholic steatohepatitis, Awareness, Practice guideline, Diagnosis, Disease management, Primary care physicians

## Abstract

**Background:**

The hepatic manifestation of metabolic syndrome is nonalcoholic fatty liver disease. Patients with nonalcoholic steatohepatitis, the progressive form of nonalcoholic fatty liver disease, have increased risk of fibrosis, cirrhosis and end-stage liver disease. Estimates of prevalence in the United States range from 20–30 % for nonalcoholic fatty liver disease and 2–5 % for nonalcoholic steatohepatitis; however, physician awareness of these diseases is limited. The purpose of this study was to determine the current level of physician awareness and practices in the diagnosis and management of nonalcoholic fatty liver disease and nonalcoholic steatohepatitis within the United States.

**Methods:**

Physicians were asked to participate in an online, 35-question survey about their awareness of various liver conditions and current practices.

**Results:**

Of the 302 responding physicians, 152 were primary care physicians, and 150 were specialists (comprised of gastroenterologists and hepatologists). More specialists than primary care physicians reported that they were aware of the differences between nonalcoholic fatty liver disease and nonalcoholic steatohepatitis (p < 0.001) and that they routinely screened for nonalcoholic fatty liver disease (p < 0.001) and nonalcoholic steatohepatitis (p < 0.001). Almost half of the responding primary care physicians reported being unfamiliar with the nonalcoholic fatty liver disease and nonalcoholic steatohepatitis differences even though they were aware of both, yet 58 % of those primary care physicians were treating patients with nonalcoholic fatty liver disease and/or nonalcoholic steatohepatitis. In addition, those primary care physicians who reported being unfamiliar with nonalcoholic steatohepatitis were treating an average of 3.7 patients and reported being as likely as familiar primary care physicians to treat new patients with nonalcoholic steatohepatitis. More than half of the specialists used noninvasive diagnostic test to confirm nonalcoholic steatohepatitis, and 10 % of the specialists reported treating patients with drugs not recommended by the current guidelines.

**Conclusions:**

Despite reporting they were not familiar with nonalcoholic steatohepatitis, primary care physicians reported they would likely continue to diagnose and manage patients with nonalcoholic steatohepatitis; therefore, more physician education on the recent practice guideline for nonalcoholic fatty liver disease and nonalcoholic steatohepatitis is needed.

## Background

Non-alcoholic fatty liver disease (NAFLD) is increasingly recognized as a major health burden in developed countries. It includes a spectrum of liver damage ranging from simple steatosis to nonalcoholic steatohepatitis (NASH), advanced fibrosis, and rarely, progression to cirrhosis [1]. In 2013, NASH became the second leading etiology of chronic liver disease among new liver transplant waitlist registrations [2]. A systematic review estimated the prevalence in the United States is about 33 % for NAFLD and 2–5 % for NASH [3]. The most recent diagnosis and management guidelines for NAFLD and NASH were published in 2012 [1]. Although it is widely accepted that a liver biopsy is the gold standard for the detection of NASH, but that it is impractical, unnecessary and cost prohibitive to perform a liver biopsy among all patients with NAFLD [4], no consensus has been reached in identifying specific biomarkers as an alternative for the diagnosing and monitoring of NAFLD-related liver disease [5]. Thus, clinical practice patterns in the diagnosing of NASH frequently diverge from published practice guidelines [6]. The lack of practical guidelines is particularly important among primary care physicians given the association of nonalcoholic fatty liver disease (NAFLD) to conditions frequently seen by these physicians, such as obesity and type 2 diabetes, and overall metabolic risk factors [1, 7, 8]. Indeed, existing data suggest there is limited awareness among general practitioners of NAFLD and its progressive form nonalcoholic steatohepatitis (NASH) [9–12].

In 2012, the American Association for the Study of Liver Disease, American College of Gastroenterology and the American Gastroenterological Association published a practice guideline for NAFLD and NASH [1]. More recently, two abstracts reported on the current practices of diagnosing and treating NASH and NAFLD using the guideline [13, 14]. Even though liver biopsy is the gold standard for diagnosing NASH, responding physicians indicated that 39 % of their NASH-diagnosed patients had not had a liver biopsy [13], and fewer than 25 % of physician respondents said they routinely performed a liver biopsy to diagnose NASH [14]. In addition, 53 % of physicians mentioned prescribing vitamin E for NASH treatment [14], even though the guideline indicates it should not be used without a liver biopsy [1], and 50 % mentioned prescribing metformin for some patients specifically to treat NASH [13] while the guidelines do not recommend its use for treating NASH [1].

Familiarity with position statements and/or the practice guideline for NAFLD or NASH may also affect NASH management. Kallman et al. [15] found that PCPs were less likely than gastroenterologists or hepatologists to be familiar with the American Association for the Study of Liver Diseases position paper on NAFLD and were more likely to defer to the American Association of Family Practice guideline. This study, however, was completed prior to the release of the practice guideline for the diagnosis and management of NAFLD in 2012 [1], and no studies have yet to be reported on whether knowledge of the practice guideline has improved confidence in treating these patients.

The prevalence of NAFLD and NASH also varies depending on the method of detection and population being studied. Using data from the United States Third National Health and Nutrition Examination Survey (NHANES III) study, overall prevalence of NAFLD excluding NASH was 16.4 % and the prevalence of NASH was 3.1 % [16]; but, the authors diagnosed NASH based on increased liver enzymes in the presence of moderate-to-severe hepatic steatosis in the absence of antibodies to hepatitis B and C and without evidence of iron overload [16]. A systematic review estimated the prevalence in the United States is about 33 % for NAFLD and 2–5 % for NASH [3]. However, these estimates were based on studies with small cohorts. In a prospective study using ultrasound as an initial screening tool and biopsy confirmation, Williams et al. [17] found that 46 % had NAFLD and 12.2 % had NASH in a cohort of active duty personnel and their families. They also found a higher prevalence of NAFLD and NASH among patients with diabetes (74 and 22.2 %, respectively) in this cohort [17]. Because of the need for liver biopsy to confirm a NASH diagnosis [1], prevalence estimates for NASH in the general population seem to be underestimated.

Together, these studies suggest that because of the increasing prevalence of obesity and underdiagnosing of related conditions such as NAFLD and NASH, there is a need for more accurate diagnosis and proper management of NAFLD. However, awareness and knowledge about NAFLD and NASH continues to be a concern that directly impacts diagnosing practices, and therefore limits our ability to accurately estimate the prevalence of NAFLD and NASH. Several recent studies have examined awareness and knowledge of NAFLD and/or NASH among different groups of healthcare workers. An Italian study of 56 general practitioners revealed 70 % underestimated the prevalence of NAFLD, and when asked only 65 % could indicate the definition of NAFLD was fatty degeneration of hepatocytes in patients with an altered glucose or lipid metabolism [12]. Wieland et al. showed that physicians, particularly PCPs, and specialists other than gastroenterologists and hepatologists within a primarily urban setting within the western United States had a low rate of recognition of NAFLD as a clinically relevant disease regarding its propensity for progression to chronic liver disease and cirrhosis and its associated morbidity and mortality [9]. They also showed that diagnostic and management approaches to patients with NAFLD were highly variable and deviated significantly from practice guidelines, indicating an overreliance on aminotransferase levels specifically to screen for NAFLD. Finally, they suggested that providers underutilized data-driven treatment modalities and also had low rates of referral to gastroenterology and hepatology subspecialists when presented with a patient with multiple metabolic risk factors and a high likelihood of having NASH histology.

In a more recent study among various specialists in a metropolitan area of Brisbane, Australia, Bergqvist et al. suggested that 75 % underestimated the prevalence of NAFLD in the general population and 89 % in high-risk populations, 57 % considered alcohol consumption to be strongly associated with NAFLD, 60 % believed simple steatosis was associated with increased liver related mortality, 66 % thought NASH could be diagnosed with liver imaging, and 71 % made no referrals to GIs/Heps for suspected NAFLD (due in part to the local healthcare system) [10]. Finally, the most recent study by Said et al. in 2013 among physicians in Wisconsin showed lack of confidence in PCPs’ knowledge about NAFLD and its management was the top barrier in treating patients with NAFLD. They also showed that, even among physicians treating high risk populations (e.g., obese, diabetics), more than half of primary care practitioners did not screen for NAFLD. The majority (84 %) also underestimated prevalence of NAFLD in the general and obese population [11].

To obtain a better understanding of awareness of and familiarity with NAFLD and NASH within an unselected group of primary care physicians within the United States, we developed and administered a nation-wide survey to primary care physicians and specialists. Additionally, we aimed to understand how levels of familiarity related to (1) current management of patients with NASH, (2) future plans for self-management or referral of patients with NASH, (3) and specific screening and diagnosing practices for NASH.

## Methods

### Survey design

A national online survey was developed and administered. United States licensed physicians were invited by mail to participate in a 15 min, online national survey. Given the purpose of this study was to determine the current level of physician awareness, familiarity, and practices in the diagnosis and management of NAFLD and NASH, the topic of the survey mentioned on the invitation was generalized to various liver conditions. Awareness of NAFLD and NASH was calculated among all physicians who entered the screener prior to having any knowledge of the purpose of the study, aside from the invitation of the study specifying it was a “national study about various conditions”. Specifically, physicians were first asked which of 11 different liver conditions they were aware of, including NAFLD, NASH, seven other liver conditions, and two sham conditions (included as a quality check). Respondents who indicated they were aware of either NAFLD and/or NASH and indicated they were willing to provide accurate responses to questions about their professional experiences qualified for the remainder of the survey. It was assumed that participation in this survey was random and represented basic interest and knowledge in liver conditions. No physician data were excluded from analysis based on their response to the screener questions.

### Ethics, consent and permissions

Physicians were offered an industry-standard honorarium for their time to complete the survey. By opting into to the survey, the respondents provided consent to use their anonymized responses to the survey questions. The study did not collect protected private health information, and it was deemed unnecessary to undergo IRB review according to national regulations [18, 19]. All survey research conducted for this article was done in accordance with the ethics practices outlined by the Council of American Survey Research Organizations (CASRO) [18].

### Survey and data collection

The survey was live from May 6, 2015 to June 4, 2015, and was comprised of 35 quantitative and qualitative questions. Quantitative questions asked about the following topics: awareness and routine screening of NAFLD and NASH (among various other liver conditions), the level of familiarity with the differences between NAFLD and NASH, current volume of patients managed for NAFLD and NASH, as well as detailed diagnosing and referral practices. Additional questions included awareness of new therapies in development for NASH and what they have heard about each. Qualitative questions asked about perceived differences between NAFLD and NASH, how physicians routinely screen for NASH and what specifically prompts them to refer patients to other physicians for NASH treatment, and how they currently manage their patients with NASH. The survey also contained a short demographic section that asked them to provide their gender, the number of years in practice, and the location and the setting of their practice.

### Data analysis

The individual identities of physician survey respondents were blinded to the study authors. All survey data were analyzed in the aggregate. Responses to the closed questions were analyzed quantitatively. All respondents who qualified for each qualitative question provided comments, because these sections of the survey were mandatory. Responses to the open-ended questions were coded into pre-selected categories before analysis. A response that addressed multiple categories was counted as multiple comments. SPSS Version 20 was used. All continuous variables were analyzed using Student’s t test. All categorical variables for which the expected cell frequencies were greater than five were analyzed using the Pearson Chi square test. A Fisher’s exact test was used whenever the expected cell frequencies were five or less. P values of less than 0.05 were considered significant.

## Results

### Survey population and responding physician demographics

A total of 8119 physicians who specialized in family or internal medicine (hereafter referred to as PCPs), 5920 gastroenterologists, and 238 hepatologists (hereafter referred to as specialists) were randomly selected from the universe of 250,838 PCPs, 14,125 gastroenterologists, and 238 hepatologists, based on the CMS National Plan and Provider Enumeration System. Out of the 354 physicians who entered the screener, 348 completed the screener. Of these 348, 344 identified themselves within our specialties of interest: 185 were PCPs, 128 were gastroenterologists, 31 were hepatologists, and 4 were specialties other than those of interest. A total of 152 PCPs, 121 gastroenterologists, and 29 hepatologists completed the screener and the remainder of the survey. All physicians who completed the screener questions qualified for the survey. This includes an additional 42 physicians (33 PCPs, 7 gastroenterologists, and 2 hepatologists) who completed the screener, but had not completed the survey at the time the data were analyzed. A comparison of the regional distribution of responding physicians (Table 1) was made with the regional distribution of the universe of PCPs and specialists, as determined by the region by state distribution designated by the Census Bureau. No significant difference between the regional breakout of the PCPs in our sample and that of the universe of PCPs was found (P = 0.080). Similarly, no difference between the regional breakout of the specialists in our sample and that of the universe of specialists was found (P = 0.502).

**Table 1:** Regional distribution of licensed physicians within the United States and who participated in the online survey

Census region	PCP		P	Specialists		P
	Sample n = 152	PCP universe n = 250,838		Sample n = 150	Universe n = 14,363	
New England	8 (6)	16,470 (7)	0.080	13 (9)	1017 (7)	P = 0.502
Middle Atlantic	26 (17)	35,366 (14)		31 (21)	2601 (18)	
East North Central	26 (17)	41,295 (16)		21 (15)	2012 (14)	
West North Central	17 (11)	18,616 (7)		5 (3)	893 (6)	
South Atlantic	37 (24)	46,588 (19)		37 (24)	2858 (20)	
East South Central	5 (3)	12,067 (5)		5 (3)	746 (5)	
West South Central	11 (7)	23,723 (9)		15 (9)	1350 (9)	
Mountain	4 (3)	16,932 (7)		7 (5)	793 (6)	
Pacific	18 (12)	39,781 (16)		16 (11)	2093 (15)	

Numbers indicated number of responding physicians within the sample (percentage of total responding physicians in the sample), and number of licensed physicians (percentage of total physicians) within each region

At the time of the survey, the responding PCPs had been practicing for a mean of 14.4 years (SD = 10.96). PCPs primarily worked in a group practice (41 %), and the majority practiced in either urban (32 %) or suburban (44 %) locations. Only 28 % of the PCPs were affiliated with an academic institution (Table 2). A total of 150 specialists (121 gastroenterologists and 29 hepatologists) completed the survey. The specialists were in practice for a mean of 16.7 years (SD = 10.69), and 126 (84 %) were male.

**Table 2:** Demographics of physician responders

	PCPs n = 152	Specialists n = 150	P
Gender			<0.001
Male	102 (67)	126 (84)	
Female	50 (33)	24 (16)	
Length of time in practice (mean years, range)	14.4 (<1–50)	16.7 (1–59)	0.087
Practice setting			<0.001
Hospital-based	36 (24)	31 (21)	
Solo private practice	29 (19)	24 (16)	
Group practice	62 (41)	93 (62)	
Clinic	25 (16)	2 (1)	
Practice location			<0.001
Urban	49 (32)	65 (43)	
Suburban	67 (44)	76 (51)	
Rural	36 (24)	9 (6)	
Affiliated with academic institution	43 (28)	51 (34)	0.284

Numbers indicate the number of respondents (percentage of total respondents)

Compared with PCPs, more specialists were male (P < 0.001), but no difference in length of time in practice was detected between the two groups (P = 0.087, Table 2). Differences in practice setting (P < 0.001) and practice location (P < 0.001) were identified. More specialists worked in a group practice (62 %) and fewer worked in clinics (1 %). Fewer specialists than PCPs practiced in rural areas (6 vs. 24 %, respectively).

### Awareness of and familiarity with NAFLD and NASH

Awareness of NAFLD and NASH was calculated among all physicians who entered the screener prior to having any knowledge of the purpose of the study, aside from the invitation of the study specifying it was a “national study about various conditions”. Specifically, physicians were first asked which of 11 different liver conditions they were aware of, including NAFLD, NASH, seven other liver conditions, and two sham conditions. Among the 344 physicians within our specialties of interest who completed the screener, 100 % indicated they were aware of NAFLD, NASH, or both. Respondents who indicated they were aware of NAFLD and NASH were then asked to indicate their familiarity with the differences between NAFLD and NASH. Among these, 49 % of the PCPs chose either not familiar or unaware of differences between NAFLD and NASH. The majority of specialists (88 %) selected extremely or very familiar with the differences between NAFLD and NASH. 11 % reported they were somewhat familiar with the differences, and 1 % indicated that they were not familiar with or unaware of these differences (Table 3). The rest indicated they were somewhat familiar with the differences. Compared with PCPs, specialists were significantly more likely to report they were familiar with the differences between NAFLD and NASH (P < 0.001). More specialists reported that they routinely screened for NASH (71 vs. 53 %; P < 0.001) and NAFLD (77 vs 56 %; P < 0.001, Table 3). There was no effect of length of time in practice on familiarity between PCPs and specialists.

**Table 3:** Physician awareness of the differences between NAFLD and NASH and current screening practices

	PCPs n = 152	Specialists n = 150	P
Awareness of NAFLD/NASH			
Familiar (extremely/very familiar)	28 (18)	132 (88)	<0.001
Somewhat familiar	51 (33)	16 (11)	
Not familiar/unaware of NASH or NAFLD	73 (49)	2 (1)	
Current screening practices			
Routinely screen for NASH	80 (53)	107 (71)	<0.001
Routinely screen for NAFLD	85 (56)	115 (77)	<0.001

Numbers indicate the number of respondents (percentage of total respondents)

When asked to specifically describe the differences between NAFLD and NASH, more PCPs than specialists reported that they thought NAFLD and NASH were the same disease (9 vs. 0 %, P = 0.001). PCPs were more likely than specialists to provide generalized descriptions of the differences between NAFLD and NASH (38 vs. 7 %, P < 0.001). Three types of responses were classified as general descriptions of the differences. These included (1) those that only mentioned NASH was more severe than NAFLD; (2) those that mentioned vague differences in the fat deposition, whether correct or incorrect (e.g., “Steatohepatitis is a disease; fatty liver is a result of cholesterol,” “One is caused by alcohol; one by too much fat in the liver”); and (3) those that mentioned only that NASH involved a rise in liver enzymes.

Compared to PCPs, specialists reported more specific differences in histologic features (51 vs. 16 %, P < 0.001, Table 4). Such responses included (1) those that mentioned the need for a liver biopsy to differentiate; (2) those that indicated the presence of ballooning in NASH; and (3) those that reported differences in histological features such as the levels of fibrosis, scarring, and/or cirrhosis. More specialists (69 %) than PCPs (24 %) indicated that NAFLD involved no liver inflammation (P < 0.001). Of note, eight PCPs (5 % of all PCPs), which included five PCPs who claimed to be familiar with NASH, and 2 % of specialists reported excessive use of alcohol as a key characteristic of NASH. Finally, 33 % of PCPs and only 1 % of the specialists reported that they were not sure of the differences between NAFLD and NASH (P < 0.001, Table 4).

**Table 4:** Coded, physician-defined differences between NAFLD and NASH

Coded responses	PCPs n = 152	Specialists n = 150	P
Generalized differences (NET)	57 (38)	10 (7)	<0.001
NASH is more severe than NAFLD	33 (22)	7 (5)	<0.001
General differences in fat deposition	10 (7)	1 (1)	0.005
NASH characterized by a rise in liver enzymes	19 (13)	2 (1)	<0.001
Thought they were the same	13 (9)	0 (0)	<0.001
NASH associated with excess alcohol intake	8 (5)	3 (2)	0.081
NAFLD involves no inflammation of the liver	45 (30)	101 (67)	<0.001
There is no rise in liver function tests associated with NAFLD, but there is with NASH	15 (10)	11 (7)	0.432
Both NASH and NAFLD can be associated with a rise in liver function tests	7 (5)	5 (3)	0.199
Increase of fat in the liver associated with both	32 (21)	41 (27)	0.202
Histologic features (NET)	24 (16)	77 (51)	<0.001
NASH is diagnosed via a liver biopsy	5 (3)	21 (14)	<0.001
Presence of ballooning indicates NASH	0 (0)	10 (7)	<0.001
Differences in levels of fibrosis/scarring/cirrhosis indicates NASH	19 (13)	45 (30)	<0.001
NASH involves damage to the liver	21 (14)	17 (11)	0.515
Not sure of the differences	50 (33)	2 (1)	<0.001

Numbers indicate the number of respondents (percentage of total respondents)

We examined the physician-defined differences based on their reported familiarity with NAFLD and NASH. Of those 79 PCPs who reported being familiar (hereafter referred to as familiar PCPs) with the differences between NAFLD and NASH in the multiple choice question, 5 (6 %) reported that they were unsure of those differences when asked to define the differences. Of 73 PCPs who were not familiar with the differences or unaware of NAFLD or NASH (hereafter referred to as unfamiliar PCPs), 22 (30 %) could provide generalized differences between the two (Table 5). Familiar PCPs were more likely than unfamiliar PCPs to correctly indicate that histologic features (P < 0.001) and inflammation (P < 0.001) were associated with NASH and not NAFLD (Table 5).

**Table 5:** Coded, physician-defined difference between NASH and NAFLD based on PCP familiarity with the NAFLD and NASH differences

Coded responses	Unfamiliar PCPs n = 73	Familiar PCPs n = 79	P
Generalized differences (NET)	22 (30)	35 (44)	0.071
NASH is more severe than NAFLD	11 (15)	22 (28)	0.076
General differences in fat deposition	2 (3)	8 (10)	0.051
NASH characterized by a rise in liver enzymes	10 (14)	8 (10)	0.496
Thought they were the same	13 (18)	0 (0)	<0.001
NASH is associated with excess alcohol intake	3 (4)	5 (6)	0.239
NAFLD involves no inflammation of the liver	11 (15)	34 (43)	<0.001
Rise in liver function tests is associated with NASH and not NAFLD	6 (8)	9 (11)	0.512
Rise in liver function tests is associated with both NASH and NAFLD	2 (3)	5 (6)	0.183
Increase of fat in the liver associated with both	5 (7)	27 (34)	<0.001
Histologic features (NET)	3 (4)	21 (27)	<0.001
NASH is diagnosed via a liver biopsy	0 (0)	5 (6)	0.036
Presence of ballooning indicates NASH	0 (0)	0 (0)	N/A
Differences in levels of fibrosis/scarring/cirrhosis indicates NASH	3 (4)	16 (20)	0.002
NASH involves damage to the liver	4 (6)	17 (22)	0.003
Not sure of the differences	45 (62)	5 (6)	<0.001

Numbers indicate the number of respondents (percentage of total respondents)

Physician may have responded with more than one coded response

### Diagnostic practices

When asked about their diagnostic practices, 50 (34 %) PCPs indicated that they diagnosed patients with NASH (Table 6), and 9 (18 %) of those could identify histological features when asked to recall the differences between NAFLD and NASH. Additionally, 18 (36 %) PCPs reported that they were not sure of any difference. In contrast 146 specialists were diagnosing patients with NASH and 75 (51 %) of those could identify differences in histological features of NASH (Table 6), and only 10 (7 %) reported generalized differences when asked to recall those differences.

**Table 6:** Current diagnostic practices of physicians and coded differences as defined by physicians who diagnose NASH

	PCPs n = 152	Specialists n = 150	P
Does the physician diagnose NASH?			
Yes	50 (34)	146 (97)	<0.001
Coded responses from physicians who diagnose NASH	n = 50	n = 146	
Generalized differences (NET)	21 (42)	10 (7)	<0.001
*NASH is more severe than NAFLD*	*13 (26)*	*7 (5)*	*<0.001*
*General differences in fat deposition*	*3 (6)*	*1 (1)*	*0.048*
*NASH characterized by a rise in liver enzymes*	*6 (12)*	*2 (1)*	*0.004*
Thought they were the same	6 (12)	0 (0)	<0.001
NASH associated with excess alcohol intake	2 (4)	3 (2)	0.272
NAFLD involves no inflammation of the liver	12 (24)	100 (69)	<0.001
Rise in liver function tests is associated with NASH and not NAFLD	5 (10)	11 (8)	0.192
Rise in liver function tests is associated with both NASH and NAFLD	4 (8)	5 (3)	0.122
Increase of fat in the liver is associated with both NASH and NAFLD	9 (18)	41 (28)	0.158
Histologic features (NET)	9 (18)	75 (51)	<0.001
*NASH is diagnosed via a liver biopsy*	*1 (2)*	*20 (14)*	*0.012*
*Presence of ballooning indicates NASH*	*0 (0)*	*10 (7)*	*0.048*
*Differences in levels of fibrosis/scarring/cirrhosis indicates NASH*	*8 (16)*	*45 (31)*	*0.042*
NASH involves damage to the liver	5 (10)	16 (11)	0.208
Not sure of the differences	18 (36)	1 (1)	<0.001

Numbers indicate the number of respondents (percentage of total respondents)

The results in italics above are the individual items that qualify to be counted towards the corresponding NET categories above them. In other words, physicians who mentioned more than one of the items in italics is counted only once in the NET category above it

Of the 44 responding PCPs who diagnosed patients with NASH, only 25 % of their patients had had a liver biopsy (Table 7). Of the 48 PCPs who had NASH patients that were diagnosed or referred by another physician, 44 % of their patients had had a liver biopsy. Those responding PCPs whose patients were diagnosed by another physician indicated that NASH diagnosis was confirmed by using noninvasive techniques, mainly ultrasound (74 %) and computed tomography scan (27 %, Table 7). Of the 150 specialists, 143 (97 %) said they diagnosed NASH (Table 7). Of these 143 specialists, 61 % of their patients had had a liver biopsy, and 63 % of their patients without a liver biopsy were not recommended one (Table 7). Among the 32 PCPs and 76 specialists who personally diagnosed at least one patient by a method other than a liver biopsy, significantly more PCPs (94 %) than specialists (50 %) reported relying solely on imaging and/or liver function tests as a primary method to diagnose NASH (P < 0.001, Table 8). Only specialists reported using FibroScan/transient elastography, and/or FibroSure as a primary method to diagnose NASH (P < 0.001).

**Table 7:** Aggregate patient-level responses by physicians and mean number of patients seen by physicians who either diagnosed or did not diagnose NASH

	PCPs	Specialists	P
Does the responding physician diagnose NASH?			
Yes	n = 44	n = 143	
Patients diagnosed with liver biopsy	25 % (4.8)	61 % (16.3)	0.001
Patients diagnosed without liver biopsy	75 % (14.5)	39 % (10.5)	0.352
No	n = 48	n = 79	
Patients had liver biopsy	44 % (4.1)	43 % (5.8)	0.320
Patients did not have liver biopsy	42 % (3.9)	48 % (6.5)	0.265
Unknown biopsy status	14 % (1.3)	8 % (1.3)	0.875
What percentage of patients who did not have a liver biopsy had their diagnosis for NASH confirmed primarily using the following method(s)?	n = 32	n = 76	
Ultrasound	74 % (14.8)	66 % (13.1)	0.754
CT scan	27 % (5.3)	20 % (4.0)	0.386
MRI	3 % (0.6)	13 % (2.6)	0.101
Proton magnetic resonance spectroscopy	2 % (0.4)	2 % (0.3)	0.914
Other	2 % (0.5)	19 % (3.8)	0.152
What is the main reason for lack of liver biopsy?	n = 32	n = 76	
Patient refusal	51 % (10.2)	33 % (6.5)	0.384
Had alternate reason	5 % (1.0)	4 % (0.8)	0.797
Not recommended a liver biopsy	44 % (8.7)	63 % (12.4)	0.394

Number of responding physicians (n) indicated patient specific data (percentage of patients within practice and mean number of patients within each category)

**Table 8:** The primary method for diagnosing patients with NASH who did not have a liver biopsy

Primary method to confirm NASH other than liver biopsy	PCPs n = 32	Specialists n = 76	P
Mentioned only imaging/liver function tests	30 (94)	38 (50)	<0.001
Ultrasound	15 (47)	21 (28)	0.073
CT scan	4 (13)	10 (13)	1.000
MRI	0 (0)	6 (8)	0.176
Sonogram	1 (3)	1 (1)	0.507
Mentioned any method other than imaging/liver function tests	2 (6)	38 (50)	<0.001
Serologic studies (unspecified)	1 (3)	12 (16)	0.103
FibroScan/Transient elastography, and/or fibrosure	0 (0)	21 (28)	<0.001
Biochemical tests/risk factors (unspecified)	1 (3)	7 (9)	0.432
Ferritin	0 (0)	1 (1)	1.000
MRCP	0 (0)	1 (1)	1.000
Too vague	0 (0)	5 (7)	0.319

We examined the physician-defined differences based on whether the respondent routinely screened for NASH (Table 9). Of those 80 PCPs who routinely screened, 20 (25 %) reported that they were unsure of the differences between NAFLD and NASH, and 6 (8 %) reported that the diseases were the same. PCPs reported more generalized differences (P < 0.001) and specialists reported more histological differences (P < 0.001).

**Table 9:** Coded, physician-defined differences between NAFLD and NASH based on those who said they routinely screen for NASH

	PCPs n = 80	Specialists n = 107	P
Generalized differences (NET)	31 (39)	9 (8)	<0.001
NASH is more severe than NAFLD	21 (26)	6 (6)	<0.001
General differences in fat deposition	4 (5)	1 (1)	0.094
NASH characterized by a rise in liver enzymes	9 (11)	2 (2)	0.007
Thought they were the same	6 (8)	0 (0)	0.005
NASH associated with excess alcohol intake	7 (9)	3 (3)	0.056
NAFLD involves no inflammation of the liver	29 (36)	70 (65)	<0.001
Rise in liver function tests associated with NASH but not NAFLD	8 (10)	8 (8)	0.542
Rise in liver function tests is associated with both NASH and	12 (15)	13 (12)	0.571
Increase of fat in the liver associated with both	20 (25)	31 (29)	0.546
Histologic features (NET)	17 (21)	50 (47)	<0.001
NASH is diagnosed via a liver biopsy	5 (6)	16 (15)	0.033
Presence of ballooning indicates NASH	0 (0)	8 (8)	0.010
Differences in levels of fibrosis/scarring/cirrhosis indicates NASH	12 (15)	30 (28)	0.48
NASH involves damage to the liver	14 (18)	11 (10)	0.151
Not sure of the differences	20 (25)	2 (2)	<0.001

Numbers indicate the number of respondents (percentage of total respondents)

When asked about what prompts physicians to screen for NAFLD and NASH, specialists were more likely than PCPs to screen patients with diabetes for NAFLD and NASH (P = 0.001, Table 10). PCPs were more likely than specialists to screen patients with abdominal pain (P = 0.001).

**Table 10:** Main reasons to screen for NASH given by physicians who routinely screen for NAFLD or NASH

	PCPs n = 80	Specialists n = 107	P
NASH associated with excess alcohol use	7 (9)	3 (3)	0.056
Ruling out other conditions	3 (4)	8 (8)	0.148
Fatigue	4 (5)	3 (3)	0.222
Abnormal labs	63 (79)	78 (73)	0.358
Abdominal pain (RUQ)	19 (24)	7 (7)	0.001
Diabetes	35 (44)	73 (68)	0.001
Metabolic Syndrome	10 (13)	20 (19)	0.254
Ultrasound/CT/Imaging	33 (41)	49 (46)	0.536
Liver biopsy	8 (10)	46 (43)	<0.001
Obesity	18 (23)	38 (36)	0.055

Numbers indicate the number of respondents (percentage of total respondents)

### Management practices

At the time of taking the survey, responding PCPs were managing an average of 8.5 patients with confirmed NASH, while unfamiliar PCPs were managing an average of 3.7 patients with confirmed NASH. Specialists were managing an average of 32.2 patients with confirmed NASH (Table 11). Physicians were asked to include only patients for whom they were the primary physician making treatment decisions to manage NASH. Of the respondents, 70 % of PCPs and 98 % of specialists were currently managing NASH and/or NAFLD patients (Table 12). More specialists than PCPs reported using vitamin E and metformin to treat current NASH patients (P < 0.001 and P = 0.019, respectively). In addition, 21 % of the PCPs indicated that they would continue to manage all of their new NASH patients.

**Table 11:** Mean number of patients with confirmed or suspected NAFLD or NASH being managed by physicians

	PCPs n = 152	Unfamiliar PCPs n = 73	Specialists n = 149^a^
Confirmed NASH	8.51	3.71	32.2
NAFLD with suspected NASH (not confirmed)	10.9	6.0	41.5
NAFLD with no NASH (confirmed)	7.94	3.2	42.1
NAFLD with no suspected NASH	17.81	12.0	65.2
Suspected NAFLD	24.19	11.9	78.3

^a^One gastroenterologist who mentioned treating 1000 NAFLD with suspected NASH patients, and 800 NAFLD with no suspected NASH patients was excluded

**Table 12:** Management practices of physicians currently treating or expecting to treat new patients with NASH

	PCPs	Specialists	P (PCPs/ Specs)
Does the physician currently manage patients with NASH and/or NAFLD?	n = 152	n = 150	
Yes	106 (70)	147 (98)	<0.001
Coded current management	n = 70	n = 144	
Vitamin E	6 (9)	86 (60)	<0.001
Hepatitis vaccination	8 (11)	9 (6)	0.189
Milk thistle	0 (0)	4 (3)	0.202
Statin	5 (7)	8 (6)	0.208
Pentoxyphyllin	0 (0)	2 (1)	0.452
Metformin	1 (1)	15 (10)	0.011
Actigall	0 (0)	2 (1)	0.452
Will the physician manage new NASH patients?	n = 148	n = 150	<0.001
Yes	31 (21)	129 (86)	
Yes, but will refer some new patients	62 (42)	18 (12)	
No, will refer all new patients	55 (37)	3 (2)	

Numbers indicate the number of respondents (percentage of total responding)

We then compared management practices for PCPs based on familiarity with NASH (Table 13). At the time of the survey, 58 % (42/73) of unfamiliar PCPs were managing patients with NAFLD or NASH. Despite lack of familiarity with the differences between NAFLD and NASH, unfamiliar PCPs were as likely as familiar PCPs to report that they will manage new NASH patients (P = 0.514). When asked what symptoms would prompt a PCP to refer some patients to other physicians, 67 % (39/58) indicated they would refer for worsening laboratory values, liver function tests or abnormal transaminases.

**Table 13:** Current management practices of physicians based on familiarity with the NAFLD and NASH differences

	Unfamiliar PCPs	Familiar PCPs	P
Does the physician currently manage patients with NASH or NAFLD?	n = 73	n = 79	0.002
Yes, NASH and/or NAFLD patients	42 (58)	64 (81)	
Will the physician manage new NASH patients?	n = 69	n = 79	0.514
Yes	13 (19)	18 (23)	
Yes, but will refer some new patients	27 (39)	35 (44)	
No, will refer all new patients	29 (42)	26 (33)	

Numbers indicate the number of respondents (percentage of total responding)

### Awareness of therapies in the pipeline for treating NASH

When asked about their knowledge of drugs for treating NASH that are in the development pipeline, more specialists than PCPs were aware of any drug in development (61 vs 36 %, P < 0.001, Table 14). Fewer than 50 % of the specialists were aware of obeticholic acid while only 12 % of PCPs were aware of it.

**Table 14:** Physician awareness of therapies in development for treating NASH

	(a) PCPs n = 152	Specialists n = 150	P
Aware of any drug in development	54 (36)	91 (61)	<0.001
Obeticholic acid (OCA)	18 (12)	72 (48)	<0.001
Aramchol	7 (5)	22 (15)	0.002
GR-MD-02	5 (3)	13 (9)	0.028
Simtuzumab	9 (6)	31 (21)	<0.001
GFT505	4 (3)	10 (7)	0.056
Proscysbi	1 (1)	10 (7)	0.004
Emricasan	1 (1)	8 (5)	0.016
GCS-100	1 (1)	2 (1)	0.374
MN-001	1 (1)	5 (3)	0.090
LUM001	2 (1)	3 (2)	0.312

Numbers indicate the number of respondents (percentage of total responding)

## Discussion

Our data suggest significant gaps in physician awareness and familiarity with NAFLD and NASH in the United States. Nearly half of the responding PCPs within our study reported being unfamiliar with the differences between NAFLD and NASH, and 58 % of PCPs who were unfamiliar with these differences indicated that they were managing patients with NASH or NAFLD. In addition, these unfamiliar PCPs were just as likely as familiar PCPs to manage new NASH patients.

Because a liver biopsy is required for the diagnosis of NASH according to the current practice guideline [1], current prevalence estimates of NASH of 2–5 % may be low; not all patients may be willing to undergo a liver biopsy for confirmation. One prospective cohort study found the prevalence of NASH to be 12.2 % among its 328 participants and 29.9 % among the 134 patients with positive ultrasound for fatty liver [17]. Others reported that surveyed physicians other than hepatologists underestimate the prevalence of [9–11, 20]. This apparent lack of understanding of the prevalence of NAFLD suggests a limited knowledge of NAFLD, and as a result screening for NAFLD and NASH may be limited. Only about half of the PCPs in our study reported that they routinely screen for NAFLD and NASH.

Williams et al. reported that the prevalence of advanced NASH in untreated patients was 2.7 % (nine patients) and extrapolated their data to the general population, suggesting that more than two million middle-aged adults in the United States may have undiagnosed advanced NASH [17]. Since patients with advanced NASH have a poor prognosis [21], PCPs, who are typically the first point of contact within the United States healthcare system, not only need to be aware of NAFLD and NASH, but also need to know how to effectively manage these patients to limit disease progression.

While our study did not specifically ask PCPs if they were aware of the guideline, we did ask questions that allowed us to indirectly assess adherence to the practice guideline. Of the surveyed PCPs who personally diagnose NASH, 75 % of their patients had not been diagnosed by using the results of a liver biopsy. Additionally, PCPs who were treating referral patients for NASH reported that 42 % of their patients had not had a liver biopsy (Table 7). Our data suggest that practicing PCPs within the United States may not be using the current practice guideline to diagnose NAFLD and NASH, and these data further support the work of others [4, 22] who suggested the current practice guideline may not provide a practical approach for PCPs to diagnose NASH. Although divergence from the guidelines might be an indicator of the lack of alternative solutions to a liver biopsy, the substantial difference in practices between PCPs and specialists in light of the large gap in knowledge between PCPs and specialists indicate this is likely a result of the limited knowledge among PCPs. PCPs might choose to continue managing patients due to the belief that there is no better alternative a specialist can offer.

At the time of our survey, unfamiliar PCPs were managing about four patients with confirmed NASH (Table 11). In addition, the proportion of responses from unfamiliar PCPs was not different from the proportion of responses from the familiar PCPs when asked about their future plans to manage new NASH patients. These data are somewhat surprising, because this indicates an apparent overconfidence of the physicians who manage NASH patients but do so without NASH-specific knowledge.

PCPs were less likely than specialists to use vitamin E to treat patients with NASH; however, we did not ask whether these PCPs only used vitamin E in patients with liver biopsy-confirmed NASH. Furthermore, some specialists were using metformin even though no data support its use for treating NASH [1]. These data indicate that these responding physicians appear to not be following the practice guideline for treating NASH. Although this could indicate lack of awareness of such guidelines, it is likely more of a reflection of the skepticism with guidelines as it relates to treatments, given their overall lack of efficacy. As expected, more specialists than PCPs did have some knowledge about the drugs in development to treat NASH.

One practical approach to narrowing the gap in knowledge between PCPs and specialists starts with establishing a program for accreditation of centers with special expertise in NAFLD-related liver conditions, particularly NASH, to raise the overall quality of care and outcomes in patients with this life-threatening disease. This program should be multidisciplinary and global in order to capitalize on the highly interrelated nature of this condition to its underlying risk factors. Efforts to create this program can mirror those of The Pulmonary Hypertension Association’s Scientific Leadership Council’s effort in establishing the PHA-Accredited PH Care Centers (PHCC) in order to raise the overall quality of care and outcomes in patients with pulmonary hypertension (PH), in particular pulmonary arterial hypertension (PAH) [23]. Similar to NAFLD, PH is a highly prevalent condition that is often confused with related conditions, with PAH affecting only a small subset of patients with a very different prognosis, similar to NASH. Like NASH, PAH was often misdiagnosed due to the need for a right heart catheterization in order to accurately determine the diagnosis. Right heart catheterizations, however, presented a challenge for many physicians and patients due to similar reasons liver biopsies will remain a challenge to perform among all patients with suspected NAFLD or NASH. Such expert center would lead the process of coming to a consensus on what biomarkers can be used in diagnosing and in evaluating drugs in development. Once revised guidelines are achieved based on these recommendations, it would serve to accredit physicians or centers in the proper diagnosing of NAFLD-related liver conditions. Additionally, it will be important for these programs to then collaborate with the highly structured existing centers, such as diabetes education centers, that are aimed to maximize knowledge and proper treatment of underlying risk factors of NAFLD-related liver conditions. Our study had three limitations. First, while low, our response rates are similar to other mailed surveys. Second, the abilities of the physicians to recall specific patient data may have influenced their responses. Third, the survey did not specifically ask about awareness of the practice guideline for NAFLD and NASH, therefore, we cannot comment on the specific knowledge of the practice guideline in relation to the diagnosis and management of NASH.

## Conclusions

The awareness of and familiarity with the differences between NAFLD and NASH by surveyed PCP in the United States is limited. While specialists were generally more knowledgeable about these differences, not all specialists were following the diagnosis and treatment guideline. Therefore, more education for PCPs and specialists is needed to ensure NAFLD and NASH are appropriately diagnosed and managed.

## Abbreviations

NAFLD: nonalcoholic fatty liver disease
NASH: nonalcoholic steatohepatitis
NHANES III: Third National Health and Nutrition Examination Survey
PCP: primary care physician
